# L161982 alleviates collagen-induced arthritis in mice by increasing Treg cells and down-regulating Interleukin-17 and monocyte-chemoattractant protein-1 levels

**DOI:** 10.1186/s12891-017-1819-3

**Published:** 2017-11-16

**Authors:** Liang Chen, Xianglei Wu, Jun Zhong, Dongqing Li

**Affiliations:** 1 Department of Orthopedics, Renmin Hospital of Wuhan University, 9 Zhangzhidong Street, Wuhan, Hubei 430060 People’s Republic of China; 2 Laboratory of Immunology, University of Lorraine, Avenue du Morvan, 54511 Vandoeuvre lès Nancy, Nancy, France; 3 Department of Microbiology, School of Basic Medical Science, Wuhan University, 185 Donghu Road, Wuhan, Hubei 430071 People’s Republic of China

**Keywords:** Collagen-induced arthritis, Interleukin-17, Monocyte chemotactic protein-1, EP4 antagonist, Rheumatoid arthritis

## Abstract

**Background:**

To investigate the effects and potential mechanism of L161982 (a kind of EP4 antagonist) on the collagen-induced arthritis (CIA) mice model.

**Methods:**

The CIA mice model were first established by immunizing with Chicken Type II Collagen on DBA/1 mice. The CIA groups were administered once a day for 2 weeks with either 5 mg/kg L161982 by intraperitoneal injections (IP), 200 U celecoxib by intragastrical injections, or 100 μl PBS (IP). At the end of the study, total arthritis score and histopathologic examination were assessed to determine CIA severity. The plasma and tissue expressions of IL-17 and monocyte chemoattractant protein-1 (MCP-1) were detected by enzyme-linked immunosorbent assay (ELISA) and Immunohistochemical staining (IHC) respectively; The number of CD4^+^CD25^+^Foxp3^+^ regulatory T cells (Treg) determined as a proportion of total CD4^+^ cells in the lymph nodes and spleen. We also tested the proliferation of isolated Tregs and the ratio of Th17 polarization of Naïve T cells under the treatment of L161982 by BrdU assay and flow cytometry respectively.

**Results:**

CIA mice treated with L161982 showed reduced arthritis scores, joint swellings, cracked cartilage surface, and less hyperplasia in the connective tissue of the articular cavity. Plasma and tissue IL-17 and MCP-1 decreased, while the proportion of Treg cells is increased both in the spleen and lymph nodes of CIA mice. Otherwise, L161982 have no direct effect on Tregs proliferation; a decreased tendency of Th17 polarization in vitro were observed in L161982-treated naïve T cells.

**Conclusion:**

Although less effective than Celecoxib, L161982 also resulted in a reduction of ankle joint inflammation in CIA mice. L161982 reduces the RA severity in CIA mice through inhibition of IL-17 and MCP-1, increasing Treg cells, and reducing inflammation. The mechanism of the reduction of IL-17 in plasma or tissue after administration of L161982 might be potentially derived from the suppression of CD4^+^ T cells differentiation into Th-17 cells.

**Electronic supplementary material:**

The online version of this article (10.1186/s12891-017-1819-3) contains supplementary material, which is available to authorized users.

## Background

Rheumatoid arthritis (RA) is a chronic debilitating autoimmune disorder that results in long-lasting joints injuries and pain [1, 2]. Various randomized controlled trials have been conducted with an aim to find an effective treatment, but some questions remain to be resolved, especially with regards to pathogenic factors, targeted compounds, or drugs [3, 4].

The activation of naïve helper T cells (Th0) can differentiate into a variety of phenotypes depending on cytokine environment: Th1, Th17, Th2, and regulatory T cells (Treg) [5]. Th1 and Th17 cells may be involved in promoting the development of RA and Treg cells may have a protective effect, while the role of Th2 cells, which are associated with immune regulation, is not fully understood [6–9].

In the pathogenesis of RA, the imbalanced secretion of cytokines results in increased inflammatory mediators, including the arachidonic acid metabolite prostaglandin E2 (PGE2) [10]. PGE2 is synthesized from arachidonic acid by cyclooxygenase (COX) and prostaglandin E synthase. PGE2 has been identified as having an immunoregulatory role in the differentiation of Th1 and Th2 cells [11, 12]. The numerous biological effects of PGE2 are mediated by four G protein-coupled receptors (EP1, 2, 3, 4). The activation of the EP4 receptors [10] may promote both inflammatory or anti-inflammatory effects, with an inflammatory role in Th17 cell-dependent diseases [13]. Of note, EP4 knock-out mice have been found to be resistant to type-II collagen antibody-induced arthritis [14].

In this paper, we investigated the effects of blocking PGE2 signaling using an EP4-receptor antagonist on disease severity in a mouse model of CIA. Additionally, we examined changes in the cytokines IL-17 and MCP-1 and resulting changes in the proportions of regulatory T cell, through which EP4-receptor antagonism can potentially modulate CIA disease progression.

## Methods

### Animals and reagents

Female DBA/1 mice of 6 to 8 weeks old were provided by the Center for Animal Experiment and ABSL-3 Laboratory, Wuhan University School of Medicine, Hubei, China. The following reagents were used in this study: EP4 receptor antagonist L161982 (*N*-[[4′-[[3-Butyl-1,5-dihydro-5-oxo-1-[2-(trifluoromethyl)phenyl]-4*H*-1,2,4-triazol-4-yl]methyl] [1,1′-biphenyl]-2-yl]sulfonyl]-3-methyl-2-thiophenecarboxamide) (Tocris, UK); Chicken Type II Collagen (Sigma, USA); Celecoxib, PGE2 and BrdU Cell Proliferation ELISA Kit (Abcam, USA); Dimethyl sulfoxide (DMSO, Sigma, USA); Freund’s Complete Adjuvant (Chondrex, USA); Interleukin-17 (IL-17), monocyte chemoattractant protein-1 (MCP-1), and ELISA kit (eBioscience, USA); Mouse antibodies: FITC-anti-CD4, PE-anti-CD25, PeCY5-anti-Foxp3, PE-CD62L and PeCY5-anti-IL-17, anti-IL-17, anti-MCP-1, anti-cleaved-caspas 3, soluble anti-CD3 and soluble anti-CD28 (eBioscience, USA); EasySep mouse CD4^+^CD62L^+^ naïve T cells isolation kit (Milenyi biotec, USA); Th17 cells inducement: mouse TGF-β1, IL-6, IL-23, anti-IFNγ and anti-IL-4 (Milenyi biotec, USA).

### Establishment of CIA mouse model and dosing regimen

Forty mice were randomly divided into five groups, 8 mice per group: the control group and four CIA groups. For CIA groups, 200 μg of chicken type II collagen dissolved in DMSO was mixed with an equal volume of Freund’s Complete Adjuvant and emulsified in ice bath. Of this emulsion, 100 μl was administrated through intradermal injection at the base of the tail and this immunization was boosted 3 weeks later [15]. For the model control group, the emulsion with Freund’s Complete Adjuvant but without chicken type II collagen was injected according to the same protocol. For the remaining CIA treatment groups, mice were treated firstly as the CIA group and then administered with 5 mg/kg of L161982 by intraperitoneal injections (IP), 200 U celecoxib by intragastrical injections (IA) or 100 μl PBS (IP) respectively [16, 17] as the previous studies. All injections were administered once per day for 2 weeks.

### Evaluation of arthritis lesions

The degree of arthritis was evaluated using scores from 0 to 4 points per foot, with a maximum of 16 points as the total arthritis score (AS) of four feet. 0 point: no joint swelling; 1 point: detectable swelling in one or more toe joints; 2 points: swelling in toe and tarsometatarsal joints; 3 points: swelling inferior the ankle line; 4 points: swelling of the entire paw or ankylosis. These evaluations were conducted on day 35 after the first immunization.

### Pathological evaluation of lesion severity on mouse model of arthritis

Mice were sacrificed by CO2 asphyxiation at the peak phase of pathological changes (34 days after the primary immunization) and the hind limbs, the ankle joints, and the toes were taken. Tissues were fixed for 2 days in 10% neutral formalin, decalcification was carried out using ethylenediaminetetraacetic acid (EDTA), and then paraffin-cut sections were stained with hematoxylin and eosin and assessed for pathological evaluation. Synovitis score was evaluated by following criteria: Grade 0 = absence of inflammatory lesions; Grade 1 = mild focal infiltrations; Grade 2 = moderate infiltrations; Grade 3 = severe infiltrations but no cartilage damages or pannus formations; Grade 4 = extremely serious inflammatory infiltrations, pannus formations and/or cartilage damages.

### Elisa

After the sacrifice, the eyeballs of mice were quickly removed and blood was collected from the orbital sinus. The quantitative measurements of IL-17 and MCP-1 levels in plasma were detected according to commercial ELISA kit protocol.

### Immunohistochemical staining

Immunohistochemical staining was performed on slides. Briefly, the slides were deparaffinized, blocked with hydrogen peroxide at the concentration of 3% and antigen retrieval was then performed in in a steam cooker for 1.5 min in 1 mM EDTA, pH 9.0. Mice IL-17, MCP-1 and Caspase 3 monoclonal antibody were applied at 1:150 diluents for 1 h. The HRP (horseradishpero xidase) labeled secondary antibody was applied for 15 min. Diaminobenzidine was used as chromogens and slides were counterstained with haematoxylin before mounting.

### Flow cytometry analysis

To test the percentage of Treg cells in vivo, mice spleen and inguinal draining lymph nodes were dissected under sterile conditions, rinsed by phosphate buffered saline (PBS), polished using a 200 mesh, and then harvested with a 30 μm filter. Approximately 1 × 10^6^ cells were stained with the mosue FITC-CD4, PE-CD25, PeCY5-Foxp3 antibodies in together; To assess Th17 cells polarization level in vitro, naïve T cells were first isolated from mice splenocytes by using of the aforementioned CD4 + CD62L+ naïve T cells isolation kit, and then cultured in the presence of cytokines including 10 ng/ml TGF-β1, 80 ng/ml IL-6, 20 ng/ml IL-23, 10 μg/ml anti-IFNγ, 10 μg/ml anti-IL-4 and 700 pg/ml PGE2 with or without 150 pg/ml of L161982. Seven days after incubation, Cells were stained with mouse FITC-anti-CD4, PE-CD62L and PeCY5-anti-IL-17 after fixation/perm to test Th17 cells polarization. All analysis were perforemed on a Gallios™ Flow Cytometer (Beckman Coulter, USA). Data were analyzed with Kaluza® software (Beckman Coulter, USA).

### In vitro proliferation assay

CD4^+^CD25^+^ cells were purified from mice splenocytes by using the CD4^+^CD25^+^ Regulatory T Cell Isolation Kit by a negative selection procedure. Cells were cultured at a density of 2 x 10^3^cells per well, and were stimulated with 0.5 μg/ml soluble mouse anti-CD3, 1 μg/ml anti-CD28 and 700 pg/ml PEG2 with or without 150 pg/ml L161982. After five days incubation at 37 ° C and 5% CO2, the cells were pulsed with BrdU (100 μM) and were assessed for BrdU incorporation 4 h later. Results are expressed as optical density (OD) at 405 nm.

### Statistical analysis

Statistical analysis was performed using SigmaPlot Version 12 (Systat Software Inc., USA). All measured variables were normally distributed. Data is presented as mean ± standard deviation. Multiple group means were tested firstly for homoscedasticity and were then compared using one-way analysis of variance (one-way ANOVA). Where significant effects were reported, post hoc evaluations were performed using Tukey’s Honestly Significant Difference (HSD) test. *P* values less than 0.05 were considered statistically significant. All statistical analyses were completed using SPSS statistical package (SPSS Inc.).

## Results

### Incidence of CIA

Three weeks after primary immunization, total 24 mice in 35 collagen induced mice were showed joint swelling with progressive worsening of symptoms compared to mice in the model- control group.The incidence of CIA is 69%. Arthritis score on day 35 were showed in Table 1.

**Table 1:** Arthritis score, plasma cytokines, and Treg proportions in CIA mice in response to treatment with celecoxib or L161982

	Mouse groups (*n* = 6)				
	Model Control	CIA-model			
		Blank	PBS	Celecoxib	L161982
Arthritis score(day 35)	0	7.00 ± 2.90	6.67 ± 2.34	3.67 ± 1.21	4.83 ± 1.17
Synovitis score	0	3.50 ± 0.54	3.83 ± 1.17	1.56 ± 0.8	1.83 ± 0.75
Il-17 (pg/ml)	14.38 ± 3.2	27.51 ± 8.1*	28.51 ± 6.4	10.65 ± 4.8^#^	13.52 ± 3.9^#^
MCP-1 (pg/ml)	13.1 ± 2.8	30.2 ± 3.4*	30.2 ± 3.4	14.98 ± 3.8^#^	15.80 ± 2.1^#^
Treg/ CD4+ T cell (%)					
Lymph node	4.7 ± 0.5	3.31 ± 0.36*	3.01 ± 0.96	3.25 ± 1.6	4.21 ± 0.52^#^
Spleen	2.8 ± 0.5	1.67 ± 0.14*	1.7 ± 0.33	1.75 ± 0.73	2.63 ± 0.41^#^

Data are mean ± SD; “CIA” means collagen induced arthritis; “Blank” means CIA model without any drug treatment; “PBS” means phosphate buffered saline. “*” means *p* < 0.01 compared to Model control group; “#” means *p* < 0.01 compared to PBS group; Additional file 1

### L161982 treatment reduced arthritis lesions and lesion progression in CIA mice

**Fig. 1:**
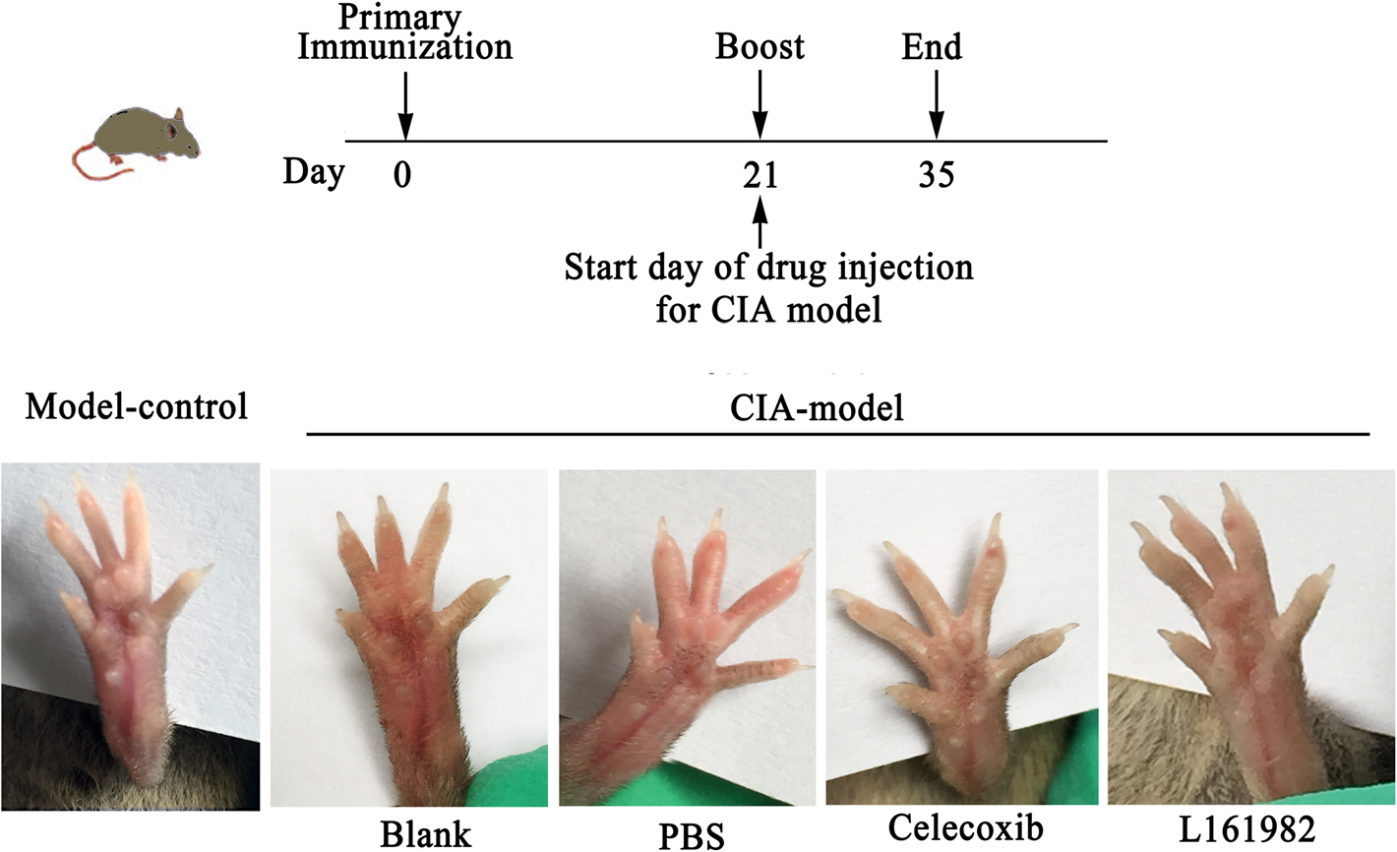
Macroscopic observations of the redness and swelling of toes 35 days after the first immunization. Upper: Time and immuniaztion of the animal experiment. Bottom: comparing with model-control group, CIA-model groups presented all redness and swelling inferior the ankle joint; Within CIA-model groups, L161982 and celecoxib group just showed less red and swollen than blank and PBS groups. The number in the upright of each figure means the arthritis score (AS) of the mouse

**Fig. 2:**
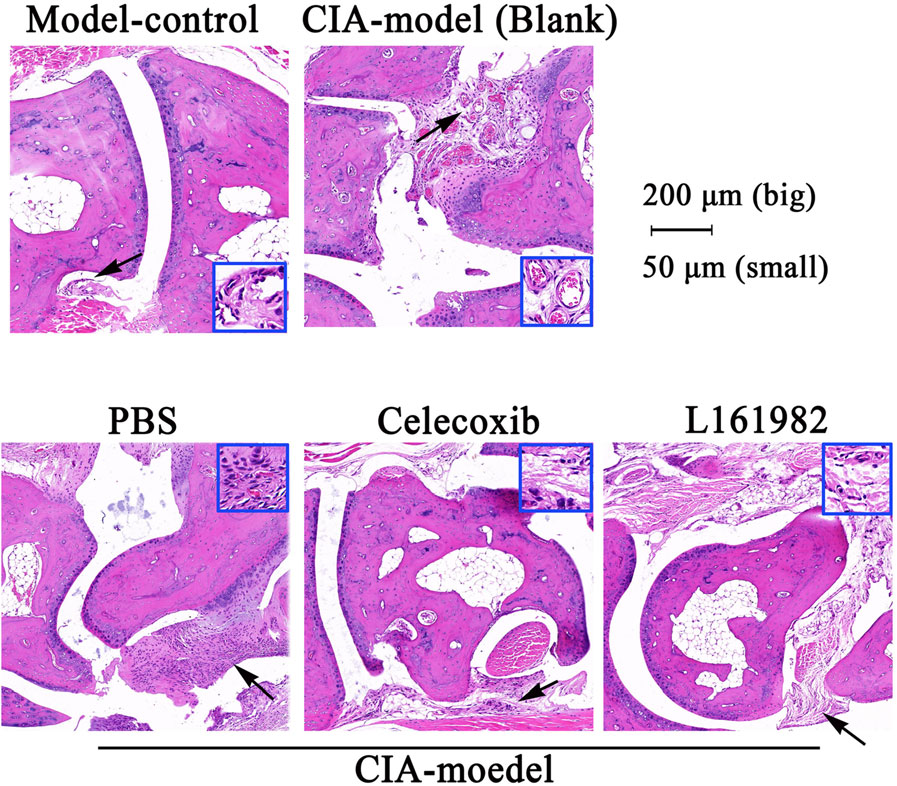
Histological examination of mice hind paws. Blank CIA mice and PBS treated CIA mice showed hyperplasia of the synovial tissue, increased new blood vessels. Increased inflammatory cells infiltration, and cracked and denudated cartilage. Celecoxib and L161982 treated CIA mice showed less hyperplasia and inflammatory cells infiltration. Synovial hyperplasia in the articular cavity is marked in black arrows. Small maps (Magnification 50 μm) within each larger picture (Magnification 200 μm) highlight areas indicated by arrows. Slides were hematoxylin and eosin stained and magnified at 200 μm/50 μm)

CIA mice were treated with L161982 showed less joint swelling and lower AS after 35 days post immunization compared with the PBS-treated mice (*P* < 0.01) and blank CIA mice (*P* < 0.01) (Fig. 1). However, the reduction in joint swelling was greater in celecoxib-treated mice than L161982-treated mice. Anatomopathological analysis showed that CIA mice treated with L161982 presented with significantly less inflammatory cells infiltration, tissue necrosis, and joint swellings in comparison to blank and PBS-treated mice (Fig. 2). These results showed that the CIA immunization model successfully produced arthritis in these DBA/1 mice.

### L161982 reduced plasma and tissue IL-17 and MCP-1 expression in CIA mice

**Fig. 3:**
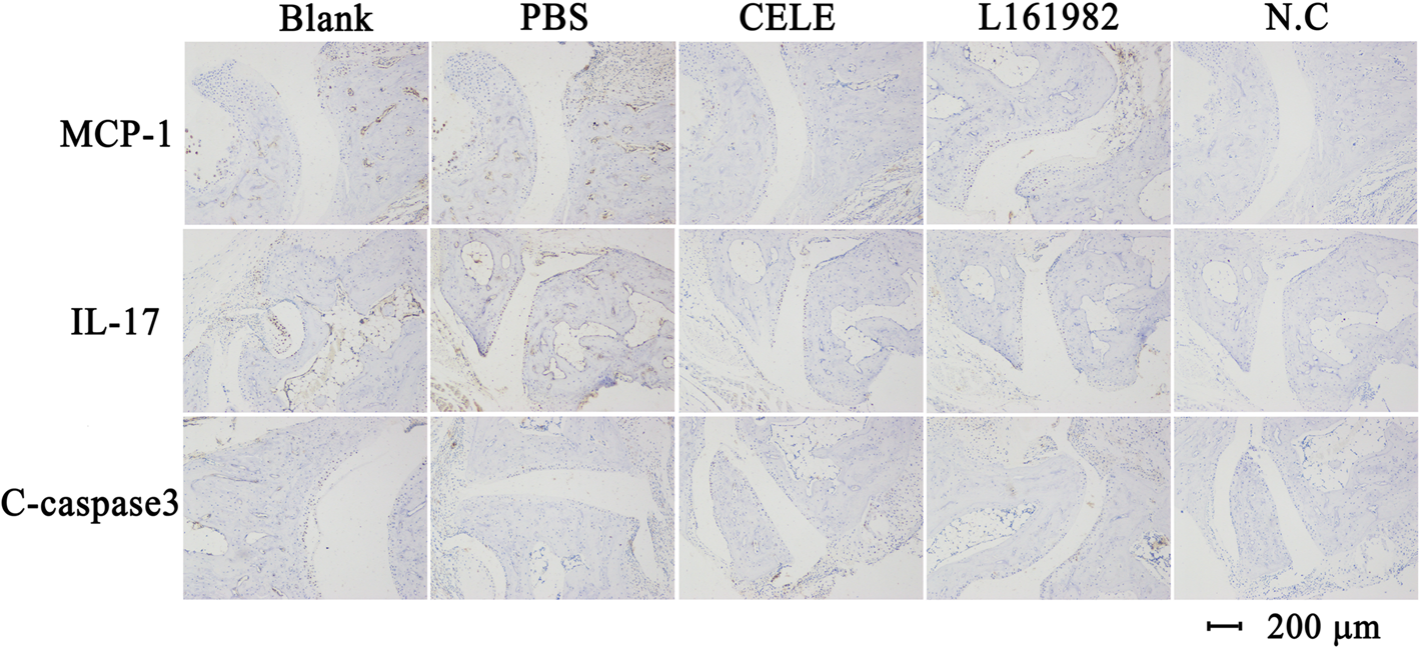
Immunohistochemical staining of CIA-model mice for testing the tissue expression of MCP-1,IL-17 and C-caspase 3. Celecoxib and L161982 treated CIA mice showed lower level of MCP-1 and IL-17 than the blank and PBS group. The expression of C-caspase 3 in all groups were looked no difference. “N.C” i.e. negative control, means tissue were stained without the corresponding primary antibody. C-caspase 3 means cleaved caspase 3

Plasma IL-17 and MCP-1 increased (*p* < 0.01) in the CIA mice compared to the CIA control mice (Table 1). In comparison to the PBS treated mice, plasma IL-17 and MCP-1 was significantly reduced in L161982-treated mice. Celecoxib-treated mice showed a greater reduction in IL-17 and MCP-1 than L161982-treated mice (Table 1). IHC staining for IL-17 and MCP-1 on tissue section confirmed our findings from plasma ELISA (Fig.3). More importantly, Cleaved-caspase-3 immunohistochemical staining of each group showed the tissues in each group have the similar cell apoptosis level (Fig.3).

### L161982 increased the ratio of Treg cells in CIA mice but could not promote the growth of Treg cells directly

**Fig. 4:**
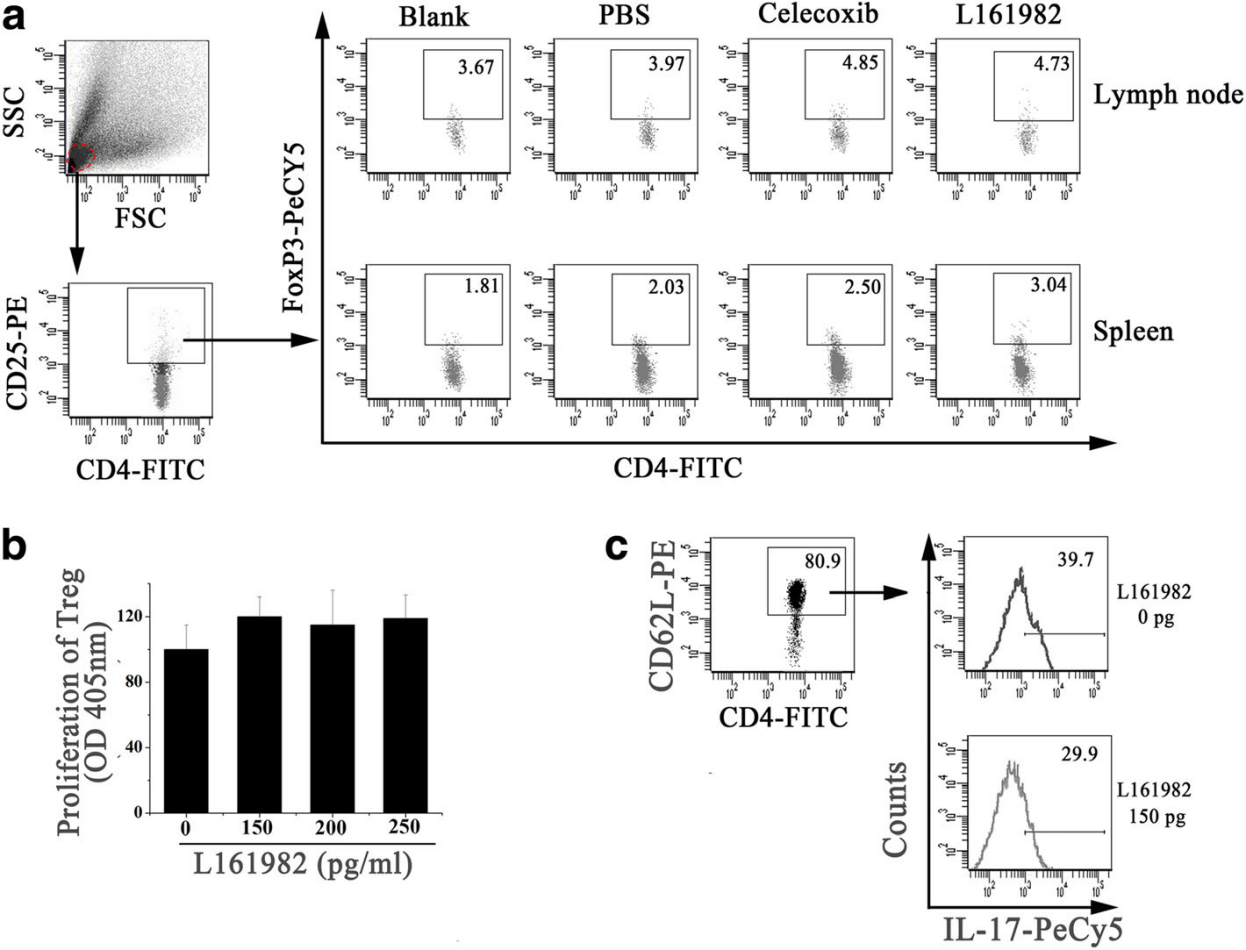
The effects of L-161982 on Treg cells and Th17cells. Fig 4a, Flow cytometry analysis showed as well as celecoxib, L-161982 increase the proportion of CD4^+^CD25^+^Foxp3^+^ Treg cells both in spleen and lymph node of the CIA-model mice; Fig. 4b, L161982 could not affect the proliferation of Treg cells in vitro. Purified CD4^+^CD25^+^ cells were stimulated with anti-CD3, anti-CD28 and PEG2 with or without L161982, then cells were mixed with BrdU for proliferation analysis as described in mothed; Fig. 4c, Naïve T cells were isolated from mouse spleen and stimulated with cytokines for differentiating into Th17 cells. The ratio of Th17 cells were decreased by treating with L161982. Additional file 1

The proportion of CD4^+^CD25^+^Foxp3^+^ Treg cells among the CD4^+^ T lymphocytes from the lymph nodes and spleen was significantly increased in L161982-treated mice compared to CIA mice, PBS-treated mice, and celecoxib-treated mice (Table 1, Fig. 4a). However, L161982 could not enhance the proliferation of purified Treg cells directly in vitro (Fig. 4b).

### L161982 suppress Th 17 differentiation of Naïve T cells in vitro

To explore the mechanism of the lower IL-17 level in CIA mice. Purified CD4^+^CD62L^+^ naïve T cells were cultured in vitro with the treatment of L161982. By measuring the ratio of IL-17 produced cell, we found Th17 cells polarization were reduced by L161982 (Fig. 4c).

## Discussion

In this study, the EP4 antagonist L161982 were uesd in treating a classic CIA model [18] to reduce arthritis scores, joint swellings, and cracked cartilage surface of the mice. All pathological feature in vivo were consistent with a previous report that L161982 could mitigate connective tissue inflammation through the inhibition of PGE2-EP4 signaling [19].

EP4 is one of the PGE2 receptor sub-types, which is a G-protein-coupled receptor involved in reproductive system and expressed on the cell surface of macrophages [20]. PGE2 receptor antagonists have been studied in many fields, including tumor and some pain treatments, and have the potential to suppress tumor-associated lymph angiogenesis and, consequently, lymphatic metastasis in breast cancer [21, 22]. Furthermore, co-administration of EP4 antagonists CJ-023423 and RO3244019 also showed an analgesic effect in a rat model [23]. This also indicated that EP4 antagonists may be therapeutically useful for RA, but the specific EP4 receptor antagonist L161982 still lacks evidence.

Although the pathological mechanisms in RA are not fully understood, dysfunction and imbalance of T-cell types are important factors. PGE2-EP4 signaling has been shown to promote inflammation through Th1 differentiation and Th17 cell expansion [24]. EP4 receptor antagonists may help alleviate RA symptoms including pain and tissue damage, potentially due to the inhibition of Th17 cell differentiation and reduced cytokine expression [25, 26]. While previous evidence has suggested that EP4 receptor antagonists may be beneficial in the CIA mouse, the mechanism of action is currently not well understood [27, 28].

Here we show that the plasma IL-17, secreted principally by Th17 cells, was higher in the CIA mice compared with the control mice, while plasma IL-17 was reduced in L161982-treated mice compared to PBS-treated mice. We further confirm this results by testing tissue IL-17 level via immunohistochemistry staining. The alleviations of RA symptoms resulting from L161982 administration were related to reductions in IL-17 [29, 30]. Low concentrations of PGE2 can increase IL-17A expression through binding to EP4 receptors and activation of EP4-cAMP signaling pathways [31]. We also observed a decreased tendency of Th17 polarization in naïve T cells in vitro, it indicates that the reduction of IL-17 in plasma or tissue after administration of L161982 might be potentially derived from the suppression of CD4^+^ T cell differentiation into Th-17 cells.

MCP-1 is derived principally from endothelial cells, fibroblasts, and monocytes, which can bind to a variety of chemokine receptors to induce lymphocyte differentiation. A previous study reported MCP-1 is an important indicator for evaluating RA disease activity [32]. Increased MCP-I level in rheumatoid arthritis is prone to endothelial dysfunction which is also indicate poor prognosis of disease [33]. Our results are compatible with these studies, however, whether the effects of L161892 on CIA are primarily by MCP-1 or other important cytokines need to be further confirmed. Importantly, higher PGE2 level in trauma can facilitate the apoptosis of cells. We verified tissue cleaved-caspase-3 level also by IHC to confirm all groups have the similar cells apoptosis level. Therefore, the reduction of IL-17 and MCP-1 are due to the effects of L161982 rather than the apoptosis of the cells.

PGE2 can also modulate the secretion of IL-12 and IL-23 through EP4 receptor activation, ultimately affecting the differentiation of CD4^+^ T cells [24]. In our study, the increased proportion of CD4^+^CD25^+^Foxp3^+^ Treg cells in L161982-treated CIA mice and BrdU incorporation assay in vitro indicated that L161982 might somehow affect the differentiation rather than proliferation of Treg cells.

Current therapeutic treatments for RA include the widely used COX-2 inhibitors, NSAIDs, and methylprednisolone. These drugs can be effective but a major limitation to their use, is the damage they cause to the gastrointestinal (GI) trac. Thus, a selective antagonist (s) of one or more critical downstream prostaglandin receptors may be more effective than broad inhibition of COX activity. Some reports had demonstrated EP4 antagonist did not cause any damage in the arthritic rat stomach, even did not worsen the gastric ulcerogenic response to stress or aspirin in normal rats. Thus, it would be an ideal therapeutic agent for the treatment of inflammatory pain [34]. However, little envidence of L161982 in related areas can be found. Therefore, the saftey of L161982 need to be further confirmed.

In short, L161982 has a beneficial effect on the pathogenesis of RA, and it might be developed as a new therapeutic treatment for RA. However, further research will be required to understand the mechanism through which L161982 acts on T cells, to identify potential side effects, and to determine the most effective therapeutic dose.

## Conclusion

Although less effective than Celecoxib, L161982 also resulted in a reduction of ankle joint inflammation in CIA mice via inhibiting IL-17 and MCP-1 expression, and increasing the ratio of Treg cells. The mechanism of the reduction of IL-17 in plasma or tissue after administration of L161982 might be potentially derived from the suppression of CD4^+^ T cells differentiation into Th-17 cells.

## Additional file

Additional file 1: Raw data. Raw data of Table 1 and Fig. 4b. (XLSX 11 kb)

## Abbreviations

AS: Arthritis score
CIA: Collagen-induced arthritis
EDTA: Ethylenediaminetetraacetic acid
ELISA: Enzyme-linked immunosorbent assay
IA: Intragastrical injections
IHC: Immunohistochemical staining
IP: Intraperitoneal injections
MCP-1: Monocyte chemoattractant protein-1
PGE2: Prostaglandin E2
RA: Rheumatoid arthritis

## References

[1] Sakkas LI, Bogdanos DP, Katsiari C, Platsoucas CD (2014). Anti-citrullinated peptides as autoantigens in rheumatoid arthritis-relevance to treatment. Autoimmun Rev.

[2] Lee HS, Woo SJ, Koh HW, Ka SO, Zhou L, Jang KY (2014). Regulation of apoptosis and inflammatory responses by insulin-like growth factor binding protein 3 in fibroblast-like synoviocytes and experimental animal models of rheumatoid arthritis. Arthritis Rheumatol..

[3] Bai F, Tian H, Niu Z, Liu M, Ren G, Yu Y (2014). Chimeric anti-IL-17 full-length monoclonal antibody is a novel potential candidate for the treatment of rheumatoid arthritis. Int J Mol Med.

[4] Boyle DL, Kim HR, Topolewski K, Bartok B, Firestein GS (2014). Novel phosphoinositide 3-kinase delta,gamma inhibitor: potent anti-inflammatory effects and joint protection in models of rheumatoid arthritis. J Pharmacol Exp Ther.

[5] Cribbs AP, Kennedy A, Penn H, Read JE, Amjadi P, Green P (2014). Treg cell function in rheumatoid arthritis is compromised by ctla-4 promoter methylation resulting in a failure to activate the indoleamine 2,3-dioxygenase pathway. Arthritis Rheumatol.

[6] Sarkar S, Fox DA (2014). Targeting IL-17 and Th17 cells in rheumatoid arthritis. Rheum Dis Clin N Am.

[7] Kosmaczewska A, Ciszak L, Swierkot J, Szteblich A, Kosciow K, Frydecka I (2015). Exogenous IL-2 controls the balance in Th1, Th17, and Treg cell distribution in patients with progressive rheumatoid arthritis treated with TNF-alpha inhibitors. Inflammation.

[8] Luo CT, Li MO (2013). Transcriptional control of regulatory T cell development and function. Trends Immunol.

[9] Kurebayashi Y, Nagai S, Ikejiri A, Koyasu S (2013). Recent advances in understanding the molecular mechanisms of the development and function of Th17 cells. Genes Cells.

[10] Tang EH, Libby P, Vanhoutte PM, Xu A (2013). Anti-inflammation therapy by activation of prostaglandin EP4 receptor in cardiovascular and other inflammatory diseases. J Cardiovasc Pharmacol.

[11] Aida T, Furukawa K, Suzuki D, Shimizu H, Yoshidome H, Ohtsuka M (2014). Preoperative immunonutrition decreases postoperative complications by modulating prostaglandin E2 production and T-cell differentiation in patients undergoing pancreatoduodenectomy. Surgery.

[12] Kowsar R, Hambruch N, Liu J, Shimizu T, Pfarrer C, Miyamoto A (2013). Regulation of innate immune function in bovine oviduct epithelial cells in culture: the homeostatic role of epithelial cells in balancing Th1/Th2 response. J Reprod Dev.

[13] Sheibanie AF, Yen JH, Khayrullina T, Emig F, Zhang M, Tuma R (2009). The proinflammatory effect of prostaglandin E2 in experimental inflammatory bowel disease is mediated through the IL-23/IL-17 axis. J Immunol.

[14] McCoy JM, Wicks JR, Audoly LP (2002). The role of prostaglandin E2 receptors in the pathogenesis of rheumatoid arthritis. J Clin Invest.

[15] Inglis JJ, Simelyte E, McCann FE, Criado G, Williams RO (2008). Protocol for the induction of arthritis in C57BL/6 mice. Nat Protoc.

[16] Yanni SE, Barnett JM, Clark ML, Penn JS (2009). The role of PGE2 receptor EP4 in pathologic ocular angiogenesis. Invest Ophthalmol Vis Sci.

[17] Chen L, Li DQ, Zhong J, XL W, Chen Q, Peng H (2011). IL-17RA aptamer-mediated repression of IL-6 inhibits synovium inflammation in a murine model of osteoarthritis. Osteoarthr Cartil.

[18] Park MJ, Park HS, HJ O, Lim JY, Yoon BY, Kim HY (2012). IL-17-deficient allogeneic bone marrow transplantation prevents the induction of collagen-induced arthritis in DBA/1J mice. Exp Mol Med.

[19] Tang EH, Cai Y, Wong CK (2015). Activation of prostaglandin E2-EP4 signaling reduces chemokine production in adipose tissue. J Lipid Res.

[20] Guo TC, Gamil AA, Koenig M, Evensen O (2015). Sequence analysis and identification of new isoform of EP4 receptors in different atlantic salmon tissues (Salmo Salar L.) and its role in PGE2 induced immunomodulation in vitro. PLoS One.

[21] Schmidt A, Sinnett-Smith J, Young S, et al. Direct growth-inhibitory effects of prostaglandin E2 in pancreatic cancer cells in vitro through an EP4/PKA-mediated mechanism. Surgery. 2017; doi:10.1016/j.surg.2016.12.037. 10.1016/j.surg.2016.12.037 PMC5433907 28222855

[22] Nandi P, Girish GV, Majumder M, Xin X, Tutunea-Fatan E, Lala PK (2017). PGE2 promotes breast cancer-associated lymphangiogenesis by activation of EP4 receptor on lymphatic endothelial cells. BMC Cancer.

[23] Sugita R, Kuwabara H, Kubota K (2016). Simultaneous inhibition of PGE2 and PGI2 signals is necessary to suppress hyperalgesia in rat inflammatory pain models. Mediat Inflamm.

[24] Bender AT, Spyvee M, Satoh T (2013). Evaluation of a candidate anti-arthritic drug using the mouse collagen antibody induced arthritis model and clinically relevant biomarkers. Am J Transl Res.

[25] Abdel-Magid AF (2014). Selective EP4 Antagonist May Be Useful in Treating Arthritis and Arthritic Pain. ACS Med Chem Lett.

[26] Duffy MM, Pindjakova J, Hanley SA, McCarthy C, Weidhofer GA, Sweeney EM (2011). Mesenchymal stem cell inhibition of T-helper 17 cell- differentiation is triggered by cell-cell contact and mediated by prostaglandin E2 via the EP4 receptor. Eur J Immunol.

[27] Chuang YC, Yoshimura N, Huang CC, Wu M, Tyagi P, Chancellor MB (2010). Expression of E-series prostaglandin (EP) receptors and urodynamic effects of an EP4 receptor antagonist on cyclophosphamide-induced overactive bladder in rats. BJU Int.

[28] Wang Y, Da G, Li H, Zheng Y (2013). Avastin exhibits therapeutic effects on collagen-induced arthritis in rat model. Inflammation.

[29] Ye L, Jiang B, Deng J, Du J, Xiong W, Guan Y, Wen Z (2015). IL-37 alleviates rheumatoid arthritis by suppressing IL-17 and IL-17-triggering cytokine production and limiting Th17 cell proliferation. J Immunol.

[30] Poloso NJ, Urquhart P, Nicolaou A, Wang J (2013). Woodward DF. PGE2 differentially regulates monocyte-derived dendritic cell cytokine responses depending on receptor usage (EP2/EP4). Mol Immunol.

[31] Lubberts E (2015). The IL-23-IL-17 axis in inflammatory arthritis. Nat Rev Rheumatol.

[32] Liou LB, Tsai WP, Chang CJ, Chao WJ, Chen MH (2013). Blood monocyte chemotactic protein-1 (MCP-1) and adapted disease activity Score28-MCP-1: favorable indicators for rheumatoid arthritis activity. PLoS One.

[33] He M, Liang X, He L, Wen W, Zhao S, Wen L (2013). Endothelial dysfunction in rheumatoid arthritis: the role of monocyte chemotactic protein-1-induced protein. Arterioscler Thromb Vasc Biol.

[34] Takeuchi K, Tanaka A, Kato S, Aihara E, Amagase K (2007). Effect of (S)-4-(1-(5-chloro-2-(4-fluorophenyoxy)benzamido)ethyl) benzoic acid (CJ-42794), a selective antagonist of prostaglandin E receptor subtype 4, on ulcerogenic and healing responses in rat gastrointestinal mucosa. J Pharmacol Exp Ther.

